# Fully Automatic Segmentation and Three-Dimensional Reconstruction of the Liver in CT Images

**DOI:** 10.1155/2018/6797102

**Published:** 2018-11-18

**Authors:** ZhenZhou Wang, Cunshan Zhang, Ticao Jiao, MingLiang Gao, Guofeng Zou

**Affiliations:** College of Electrical and Electronic Engineering, Shandong University of Technology, Zibo City 255049, China

## Abstract

Automatic segmentation and three-dimensional reconstruction of the liver is important for liver disease diagnosis and surgical treatment. However, the shape of the imaged 2D liver in each CT image changes dramatically across the slices. In all slices, the imaged 2D liver is connected with other organs, and the connected organs also vary across the slices. In many slices, the intensities of the connected organs are the same with that of the liver. All these facts make automatic segmentation of the liver in the CT image an extremely difficult task. In this paper, we propose a heuristic approach to segment the liver automatically based on multiple thresholds. The thresholds are computed based on the slope difference distribution that has been proposed and verified in the previous research. Different organs in the CT image are segmented with the automatically computed thresholds, respectively. Then, different segmentation results are combined to delineate the boundary of the liver robustly. After the boundaries of the 2D liver in all the slices are identified, they are combined to form the 3D shape of the liver with a global energy minimization function. Experimental results verified the effectiveness of all the proposed image processing algorithms in automatic and robust segmentation of the liver in CT images.

## 1. Introduction

Liver diseases have become one of the most common causes of deaths in the world. Researchers have focus on the prevention and treatment of liver diseases for many years. In recent years, computed tomography (CT) imaging has been widely used in liver disease diagnosis and surgical treatment because tumors or hepatic lesions could be observed easily from the CT image. For the captured CT images, the liver slices need to be examined in the two dimensions one by one. Consequently, it lacks an overall image of the 3D liver. Furthermore, it takes clinicians considerable time to view all the slices and diagnose the disease or evaluate the liver function based on the information divided and presented in different images. Therefore, it is desirable that 2D liver slice is segmented from CT images, and the 3D liver is reconstructed automatically and robustly beforehand. Thus, the clinicians could get the information of the 3D liver at a glance and diagnose the liver disease or evaluate the liver function more conveniently. This desire has led researchers worldwide to devote themselves to the research of coming up with automatic and robust liver segmentation methods. After so many years of research, it remains an open problem because the liver is adjacent to many other organs, such as the kidneys, spleen, stomach, intestines, and bones. In many cases, the intensity of the liver and that of the adjacent organ is indistinguishable. In addition, the shape of the liver varies according to the individuals. As a result, automatic and robust segmentation of the liver from the CT images remains as one of the most challenging artificial intelligence task for many decades.

After so many years of study and research, quite a few liver segmentation methods have been proposed, though none of them have achieved adequate accuracy so far. Among them, methods based on statistical and probabilistic models became the most popular ones [1–4]. Yet, such kinds of methods require a large size of training samples, which decreases the segmentation efficiency tremendously. Even if dictionary and sparse coding is used to reduce the redundant information, the segmentation efficiency is still not satisfactory. In recent years, deep learning and convolutional neural network have been used to segment the liver in the CT images, and the reported accuracy appears to be promising [5, 6]. Similarly, deep learning or convolutional neural network-based methods rely heavily on the training datasets to yield accurate segmentation results. In other words, the accuracy will not be acceptable if the training datasets are not similar enough to the tested case. In reality, the liver of the patients varies tremendously, and it is thus difficult to acquire a complete training datasets. In such situations, both statistical/probabilistic models-based methods and neural network-based methods might fail completely in identifying the livers of some individuals.

There are also many other methods that do not rely on training datasets. The popular segmentation methods used in identifying the boundary of the liver in CT images include active contour [7], threshold selection [8], level set [9], and graph cuts [10–12]. Since its surrounding tissues and connected organs in different slices are different, one segmentation method or several combined segmentation methods [7–12] usually could not segment the liver robustly and fully automatically. Hence, heuristic methods are proposed to segment the liver, its surrounding tissues, and its connected organs as a whole [13–15]. For instance, the surrounding tissues are segmented first and then serve as a constraint for the segmentation of the liver. In this way, the surrounding tissues are changed from interference factors to the markers of constraint. Thus, the liver could be segmented robustly and automatically [16].

In this paper, we also propose a heuristic method based on multiple thresholds selection and morphological operations. With a global threshold, we could robustly segment all the connected organs in each CT slice, such as the ribs, the spine, the heart, the kidney, and the stomach. With another global threshold, we could robustly segment the liver with the logic not operation of the segmentation result of the connected organs. Therefore, robust threshold selection becomes a critical step in the proposed method. We utilize the slope difference distribution-based threshold selection (SDDTS) method to calculate multiple thresholds in this research work. The robustness of the slope difference distribution-based threshold selection method and its advantage over state-of-the-art segmentation methods have been verified in our previous studies [17, 18]. At first, we calibrate the parameters of the slope difference distribution-based threshold selection method with several typical CT images and then use the calibrated parameters for all the CT slices. After segmentation by the global threshold, the segmentation result is filtered by minimizing the Gibbs energy function [19] to reduce inhomogeneity. Then, morphological operations [20] are used to merge divided parts of the same organ, and the merged organ is used as liver segmentation constraint. Since the intensities of the surrounding tissues and that of the liver might be completely the same, we use centroids of the segmented ribs and the spine to fit a curved line, which is then used to separate the liver and the surrounding tissues. After liver segmentation, its boundary is further refined by spline filtering.

## 2. Methods

**Figure 1:**
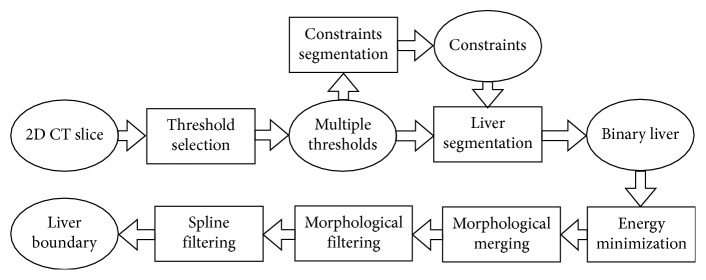
Flowchart of the proposed method.

The proposed method is heuristic, and it contains a series of image processing algorithms that vary depending on the index of the CT slice. The core image processing algorithms include (1) threshold selection based on slope difference distribution [17, 18] and image segmentation with the selected threshold; (2) energy minimization of segmentation result to eliminate noise; (3) morphological filtering; (4) morphological merging; and (5) spline filtering. The flowchart of the proposed method is shown in Figure 1. The multiple thresholds are calculated from the inputted 2D CT slice. One threshold is used for liver segmentation, and the other thresholds are used for constraint segmentation. The core image processing algorithms are used during constraint segmentation and computing the boundary of the segmented liver.

### 2.1. Threshold Selection

The slope difference distribution is computed from the histogram of the image, and it reflects the global variation rate of the histogram. With the assumption that the thresholding point between two pixel classes varies greatest, the thresholding point could be computed based on the variation rate of the histogram, i.e., the slope difference distribution. The slope difference distribution is formed by a series of slope difference that is calculated at each sampled point of the smoothed histogram. At each sampled point of the smoothed histogram, *N* points on its left are selected to fit a line model and *N* points on its right are selected to fit another line model. The slopes of these two line models are calculated. The left slope is subtracted from the right slope, and the slope difference at this sampled point is obtained. First of all, the histogram of the image needs to be calculated and smoothed as follows.

The grey-scale values of the image are scaled to the interval [1, 255], and then the histogram distribution *P*(*x*) is calculated as follows:

(1) $$\begin{array}{c} P\left(x=i\right)=\frac{F_{i}}{F_{j}},\;i=1,2,\ldots ,255,\;\;j\in \left[1,255\right], \end{array}$$

where *F*_*i*_ denotes the total number of pixels that equals to *i* and *F*_*j*_ denotes the maximum number of pixels that occurs at *j* in the interval [1, 255]. The histogram distribution, *P*(*x*), is then transformed to the frequency domain by the discrete Fourier transform (DFT):

(2) $$\begin{array}{c} F\left(k\right)=\overset{255}{\underset{x=1}{\sum }}P\left(x\right)e^{-i\left(2\pi kx/255\right)},\;k=1,2,\ldots ,255. \end{array}$$

Only the low frequencies from 0 to *W* and the symmetric frequencies from 255 − *W* to 255 are kept:

(3) $$\begin{array}{c} F^{\prime }\left(k\right)=\left\{\begin{array}{cc} F\left(k\right), & \;k=1,2,\ldots ,W,\\ F\left(k\right), & \;k=255-W,\ldots ,254,255,\\ 0, & \;k=W+1,\ldots ,255-W, \end{array}\right. \end{array}$$

where *W* is the bandwidth of the low-pass DFT filter. Its value and the value of *N* are obtained by parameter calibration [17, 18]. In general, there are always optimal values of *W* and *N* to yield the optimal threshold. However, it is still challenging to determine the optimal *W* and *N* by simple parameter calibration. When the values of *W* and *N* are not determined properly, the threshold will be determined inaccurately. The reason we choose the Fourier transform-based filter instead of other popular filters is based on the quantitative evaluation and comparison [19]. We found that the Fourier transformation-based filter outperforms other filters significantly in this conducted research work. Transforming from the frequency domain back to the spatial domain

(4) $$\begin{array}{c} P^{\prime }\left(x\right)=\frac{1}{255}\overset{255}{\underset{k=1}{\sum }}F^{\prime }\left(k\right)e^{i\left(2\pi xk/255\right)},\;x=1,2,\ldots ,255, \end{array}$$

where *P*′(*x*) is the smoothed histogram distribution.

To compute the slope difference, we fit two-line models on both sides of the sampled point. The line model is formulated as follows:

(5) $$\begin{array}{c} y_{i}=ax_{i}+b, \end{array}$$

where (*x*_*i*_, *y*_*i*_), *i*=1+*N*,…, 255 − *N*, is the sampled point on the smoothed histogram distribution and *a* is the slope of the line model. The coefficient of the line model [*a*, *b*]^T^ is computed as follows:

(6) $$\begin{array}{c} {\left[a,b\right]}^{\text{T}}={\left(B^{\text{T}}B\right)}^{-1}B^{\text{T}}Y, \end{array}$$

(7) $$\begin{array}{c} B=\left[\begin{array}{cc} x_{i+1-N} & 1\\ x_{i+2-N} & 1\\ \vdots & \vdots \\ x_{i-1} & 1\\ x_{i} & 1 \end{array}\right]\;\;\mathrm{or}\;\;\left[\begin{array}{cc} x_{i} & 1\\ x_{i+1} & 1\\ \vdots & \vdots \\ x_{i-2+N} & 1\\ x_{i-1+N} & 1 \end{array}\right], \end{array}$$

(8) $$\begin{array}{c} Y={\left[y_{i+1-N},y_{i+2-N},\ldots ,y_{i-1},y_{i}\right]}^{\text{T}}\;\;\mathrm{or}\;\;{\left[y_{i},y_{i+1},\ldots ,y_{i-2+N},y_{i-1+N}\right]}^{\text{T}}, \end{array}$$

where *B* is the design matrix of the least square fitting method and is the input data vector. Moreover, [(*x*_*i*+1−*N*_, *y*_*i*+1−*N*_), (*x*_*i*+2−*N*_, *y*_*i*+2−*N*_),…, (*x*_*i*−1_, *y*_*i*−1_), (*x*_*i*_, *y*_*i*_)] are the *N* adjacent points at the left side of the point (*x*_*i*_, *y*_*i*_), and [(*x*_*i*_, *y*_*i*_), (*x*_*i*+1_, *y*_*i*+1_),…, (*x*_*i*−2+*N*_, *y*_*i*−2+*N*_), (*x*_*i*−1+*N*_, *y*_*i*−1+*N*_)] are the *N* adjacent points at the right side of the point (*x*_*i*_, *y*_*i*_). The left slope and the right slope, *a*_l_ and *a*_r_ at point (*x*_*i*_, *y*_*i*_), are then obtained from Equation (6). The slope difference at point (*x*_*i*_, *y*_*i*_) is then computed as follows:

(9) $$\begin{array}{c} s\left(i\right)=a_{\text{r}}\left(i\right)-a_{\text{l}}\left(i\right),\;i=1+N,\ldots ,255-N. \end{array}$$

The discrete version is denoted as *s*(*i*), and its continuous version is denoted as that is named as slope difference distribution. Let the derivative of *s*(*x*) equal zero and solve it, the valleys *V*_*i*_, *i*=1,2,…, *M*, with greatest local variations are obtained. The positions where these valleys lie are the thresholds that separate different pixel classes. One fundamental property of the slope difference distribution is that the positions of the valleys change monotonically with the line model fitting parameter *N*. Hence, the parameter could be calibrated to yield the optimal threshold. After the optimum threshold *T* is selected, the image is segmented by the following equation:

(10) $$\begin{array}{c} I_{\text{b}}=\left\{\begin{array}{c} 1,\;I\left(i,j\right)\geq T,\\ 0,\;I\left(i,j\right)<T, \end{array}\right. \end{array}$$

where *I* is the original image and *I*_b_ is the binarized image. (*i*, *j*) is the index of the pixel in the image. Since multiple thresholds are needed to segment different organs, Equation (9) is the basic format of segmenting a single object. More conditions need to be added in Equation (9), when several independent segmentation results are combined to segment the liver.

### 2.2. Energy Minimization

There are many popular noise reduction methods, e.g., the discrete Fourier transformation-based filter [20] and the wavelet image processing [21]. The noise reduction methods are usually applied beforehand in the preprocessing [20]. Since we did not reduce the noise beforehand, we apply a noise reduction procedure immediately after the segmentation. The noise would cause region inhomogeneity, and this inhomogeneity could be formulated by the Gibbs distribution:

(11) $$\begin{array}{c} P\left(X=x\right)=\frac{1}{\sum_{x\in \text{L}}e^{-\sum_{\text{c}\in \text{C}}V_{\text{c}}\left(x\right)}}e^{-\sum_{\text{c}\in \text{C}}V_{\text{c}}\left(x\right)}, \end{array}$$

where *V*_c_(*x*) is the potential function associated with clique c. The clique c is defined as a set of sites such that any two elements in the clique are neighbours of each other [19]. *L* is the total number of pixel classes. *V*_c_(*x*) is defined by the following equation:

(12) $$\begin{array}{c} V_{\text{c}}\left(x\right)=\left\{\begin{array}{cc} -\beta , & \;\text{all}\;\;\mathrm{values}\;\;\mathrm{of}\;\;c\;\;\mathrm{are}\;\;\mathrm{equal,}\\ \beta , & \;\text{else,} \end{array}\right. \end{array}$$

where *β* is the constant and 1 is its default value. To reduce the noise in the binarized image, the total energy over the whole image is minimized as follows:

(13) $$\begin{array}{c} I_{\text{o}}=\underset{I_{\text{label}}\left(u,v\right)\in \left[1,M_{\text{in}}\right]}{\arg\min}\overset{M_{\text{in}}}{\underset{l=1}{\prod }}\overset{U}{\underset{u=1}{\sum }}\overset{V}{\underset{v=1}{\sum }}\underset{\text{c}\in \text{C}}{\sum }V_{\text{c}}\left(x\right), \end{array}$$

where *I*_o_ is the filtered image with Gibbs energy minimization; *u* and *v* are the pixel indexes of the image in the vertical direction and in the horizontal direction, respectively; and *U* and *V* are the resolution of the image in the vertical direction and in the horizontal direction, respectively.

### 2.3. Morphological Filtering

Besides the noise, there are many small binarized blobs that do not belong to the liver or the segmented organ. These small blobs could not be removed by energy minimization. A popular way to remove these small blobs is to count the areas of all the blobs and remove some of the blobs based on their areas morphologically. However, there are also many situations in which these small blobs are connected with the liver or the segmented organ, which make the removal of them more difficult. To remove all these interference blobs, we propose a morphological filtering method that contains the following steps.

*Step 1*. Erode the segmentation result *I*_0_ morphologically as follows:

(14) $$\begin{array}{c} I_{0}^{\prime }=I_{0}\;⊖\;B=\left\{z\;\left|{\left(B\right)}_{z}\right.\;\subseteq \;I_{0}\right\}, \end{array}$$

(15) $$\begin{array}{c} {\left(B\right)}_{z}=\left\{c\;\left|\;c=p+z,p\in B\right.\right\}, \end{array}$$

(16) $$\begin{array}{c} I_{0}=I_{0}^{\prime }, \end{array}$$

where *B* is the 4-connected structure element with the disk shape and its radius is 1, *p* is the point in the structuring element *B*, and *z* is the translation vector.

*Step 2*. Repeat Step 1 *N*_F_ times. The default value of *N*_F_ is 8.

*Step 3*. Dilate the segmentation result *I*_0_ morphologically as follows:

(17) $$\begin{array}{c} I_{0}^{\prime }=I_{0}\;⊕\;B=\left\{z\text{ |}{\left(B^{\text{s}}\right)}_{z}\;\cap \;I_{0}\neq \emptyset \right\},\\ I_{0}=I_{0}^{\prime }, \end{array}$$

where *B*^s^ denotes the symmetric or supplement of *B*.

*Step 4*. Repeat Step 3 *N*_F_ times.

In summary, the proposed morphological filtering method removes the small blobs by a repeating morphological erosion process first. Then, it restores the eroded liver or other organs by a morphological dilation process with the same repeating times.

### 2.4. Morphological Merging

On the contrary, there are situations where the segmentation result of the organ specially the stomach is split into different parts. To utilize the segmentation result more effectively, it is required that the split parts are merged into a united one. To unite these split parts, we propose a morphological merging method that contains the following steps.

*Step 1*. Dilate the segmentation result *I*_0_ morphologically as follows:

(18) $$\begin{array}{c} I_{0}^{\prime }=I_{0}\;⊕\;B=\left\{z\;\left|{\left(B^{\text{s}}\right)}_{z}\right.\;\cap \;I_{0}\neq \emptyset \right\},\\ I_{0}=I_{0}^{\prime }. \end{array}$$

*Step 2*. Repeat Step 1 *N*_M_ times. The default value of *N*_M_ is 16.

*Step 3*. Erode the segmentation result *I*_0_ morphologically as follows:

(19) $$\begin{array}{c} I_{0}^{\prime }=I_{0}\;⊖\;B=\left\{z\text{ |}{\left(B\right)}_{z}\;\subseteq \;I_{0}\right\},\\ {\left(B\right)}_{z}=\left\{c\text{ | }c=p+z,p\in B\right\},\\ I_{0}=I_{0}^{\prime }. \end{array}$$

*Step 4*. Repeat Step 3 *N*_M_ times.

As can be seen, the morphological merging is the opposite process of the morphological filtering. It connects the split parts and merges them into one united one by a repeating morphological dilation process first. Then, it restores the dilated organ by a morphological erosion process with the same repeating times.

### 2.5. Spline Filtering

After the liver is segmented, its boundary *B* is extracted first. The final smooth boundary *B*_s_ is computed by minimizing the energy between the extracted boundary *B* and the fitted polynomial spline by the following equation:

(20) $$\begin{array}{c} E\left(B_{\text{s}}\right)=\left(1-\alpha \right)\int {\left|B_{\text{s}}\left(j\right)-B\left(j\right)\right|}^{2}\;dj+\alpha \int {\left|\frac{d^{2}B_{\text{s}}\left(t\right)}{dt^{2}}\right|}^{2}\;dt, \end{array}$$

where *α* is the smoothing factor and its default value is 0.5.

### 2.6. Liver Segmentation

**Figure 2:**
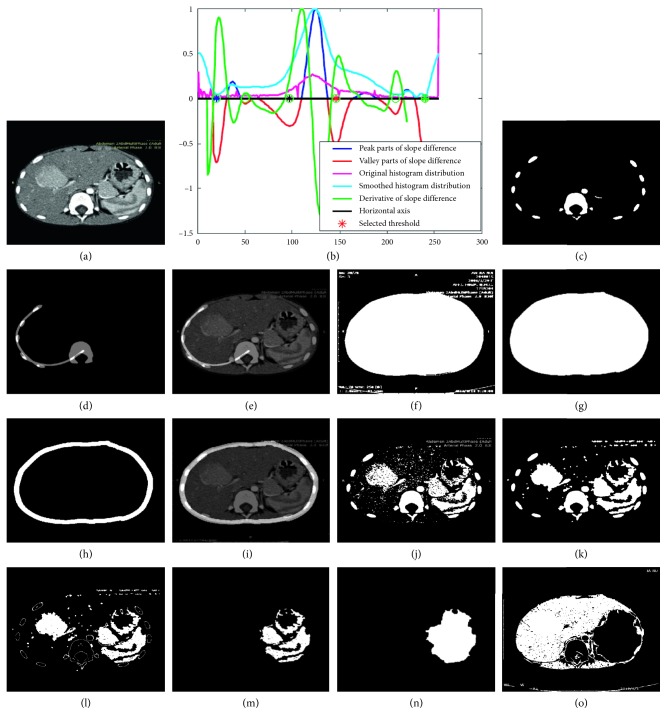
Demonstration of the proposed liver segmentation method with a typical image: (a) the original image; (b) threshold selection process by slope difference distribution; (c) the segmented ribs and spine; (d) the fitted curve based on the centroids of the ribs and the spine; (e) the fitted curve overlaying on the original image; (f) the segmented body; (g) the segmented body after morphological filtering; (h) the extracted circular part by erosion; (i) the circular part overlaying on the original image; (j) the segmented stomach; (k) the segmented stomach after energy minimization; (l) subtraction of the bones; (m) the extracted stomach after morphological filtering; (n) the merged stomach; (o) the segmented liver.

We use a typical CT image as shown in Figure 2 to illustrate the proposed segmentation method. First, the thresholds are computed with the slope difference distribution as shown in Figure 2. Four thresholds are calculated, and they are shown in Figure 2, respectively. The threshold *T*_1_ to segment the spine and ribs is denoted by the green asterisk in the green circle, the threshold *T*_2_ to segment the body is denoted by the blue asterisk in the green circle, the threshold *T*_3_ to segment the stomach is denoted by the red asterisk in the green circle, and the threshold *T*_4_ to segment the liver is denoted by the black asterisk in the green circle. With the threshold *T*_1_, the spine and the ribs are segmented as follows:

(21) $$\begin{array}{c} I_{\text{bones}}=\left\{\begin{array}{cc} 1, & \;I\left(i,j\right)\geq T_{1},\\ 0, & \;I\left(i,j\right)<T_{1}, \end{array}\right. \end{array}$$

where *I*_bones_ denotes the segmented spine and ribs, and it is shown in Figure 2. The centroids of these blobs are calculated as the means of all the pixels they contain. Based on the computed centroids, the blobs are divided into two classes, the first class of blobs that include the blobs on the left and the second class that includes the blobs on the right and around the center. The blobs in the second class are filtered based on their areas, and only the largest one (the spine) is kept. The centroids of the blobs in the first class and the largest one in the second class are used to fit a second-order curve *I*_curve_ by the least squared method (Equations (6–8)) as shown in Figure 2. This fitted curve *I*_curve_ is used to separate the tissues with almost the same intensity from the liver. Figure 2 shows the fitted curve *I*_curve_ overlaying on the original image *I*. As can be seen, the liver and its neighboring tissue on the bottom are separated successfully.

With the threshold *T*_2_, the whole body is segmented as follows:

(22) $$\begin{array}{c} I_{\text{body}}=\left\{\begin{array}{cc} 1, & \;I\left(i,j\right)\geq T_{2},\\ 0, & \;I\left(i,j\right)<T_{2}, \end{array}\right. \end{array}$$

where *I*_body_ denotes the segmented body, and it is shown in Figure 2. Figure 2 shows the segmentation result of the body after morphological filtering. As can be seen, all the small blobs are removed successfully. The filtered body is then eroded morphologically (Equations (14)–(16)) by the disk structuring element with the radius of *N*_c_. *N*_c_ is calculated as the average width of the segmented ribs, and it is 16 in this specific example. The eroded body is then subtracted from the filtered part, and the constraint circular part *I*_cir_ is obtained as shown in Figure 2. This constraint circular part is also used to eliminate the surrounding tissues that have the same or similar intensities with that of the liver. The constraint circular part overlaying on the original image is shown in Figure 2. As can be seen, it is adjacent to the liver, but almost not covering any liver part.

**Figure 3:**
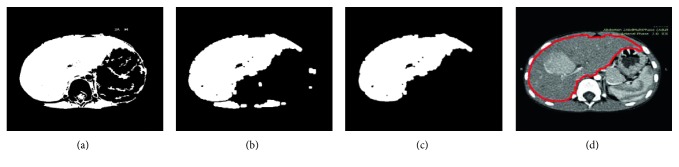
Demonstration of the proposed liver segmentation method with a typical image: (a) the segmented liver after energy minimization; (b) the segmented liver after morphological filtering; (c) the segmented liver after morphological area filtering; (d) the calculated liver boundary overlaying on the original image.

With the threshold *T*_3_, the stomach is segmented as follows:

(23) $$\begin{array}{c} I_{\text{st}}=\left\{\begin{array}{cc} 1, & \;I\left(i,j\right)\geq T_{3},\\ 0, & \;I\left(i,j\right)<T_{3}, \end{array}\right. \end{array}$$

where *I*_st_ denotes the segmented stomach, and it is shown in Figure 2. It is then filtered by energy minimization, and the result is shown in Figure 2. The filtered result is subtracted by the segmented bones *I*_bones_ shown in Figure 2, and the subtraction result is shown in Figure 2. The morphological filtering is applied on the subtraction result, and then, only the parts on the right of the spine are kept as shown in Figure 2. The morphological merging is applied on the kept parts, and the merged stomach *I*_stm_ is shown in Figure 2. With the constraint of fitted curve *I*_curve_, the circular part *I*_cir_, and the merged stomach *I*_stm_, the liver is segmented by the threshold *T*_4_ as follows:

(24) $$\begin{array}{c} I_{\text{liver}}=\left\{\begin{array}{cc} 1, & \;I\left(i,j\right)\geq T_{4}\;\&\;I_{\text{curve}}\left(i,j\right)<1\;\&\;I_{\text{cir}}\left(i,j\right)<1\;\&\;I_{\text{stm}}\left(i,j\right)<1,\\ 0, & \;\text{else,} \end{array}\right. \end{array}$$

where *I*_liver_ denotes the segmented liver, and it is shown in Figure 2. It is then filtered by energy minimization, and the filtered result is shown in Figure 3(a). As can be seen, there are still significant interference blobs. Immediately following the energy minimization, the morphological filtering is applied, and the filtering result is shown in Figure 3(b). As can be seen, the interference blobs are reduced significantly, and the left blobs could be removed by the morphological area filtering. The largest blob (the liver) is kept after the morphological area filtering and it is shown in Figure 3(c). The boundary of the segmented liver is extracted and smoothed by spline filtering.  Figure 3(d) shows the smoothed liver boundary overlaying on the original image. As can be seen, the extracted boundary matches the liver very well.

### 2.7. Three-Dimensional Reconstruction

After the two-dimensional livers in all the CT slices are segmented and their boundaries are extracted, each boundary is sampled evenly with the same number of points; i.e., the distance between any two adjacent sampled points on the same boundary is almost the same. The number of the sampling points is chosen as 200 in this study. Then, all the sampled points from all the slices are stacked together according to their practical pixel distances during the CT scanning. As a result, the three-dimensional coordinates of all the sampled points are obtained. The index of the sampled points in each slice is aligned with each other in the stacking direction (*z* direction). Then, we get 200 curved lines in the stacking direction, and the number of the points on the curved line equals the stacking number *N*_stack_. We resample the curved lines by the following spline interpolation filter:

(25) $$\begin{array}{c} P^{\text{f}}\left(m,n\right)=\underset{\text{f}}{\mathrm{argmin}}\left(\left(1-\alpha \right)\times \overset{200}{\underset{j=1}{\sum }}{\left|P\left(m,j\right)-f\left(j\right)\right|}^{2}+\alpha \times \int^{}{\left|\frac{d^{2}f\left(t\right)}{dt^{2}}\right|}^{2}\;dt\right), \end{array}$$

where *P*^f^ denotes the smoothed point and *P* denotes the original sampled point. *α* is a smoothing factor and *f* is the fitted spline function (*m*=1,2,…, *N*_stack_, *n*=1,2,…, 200).

## 3. Results and Discussion

**Figure 4:**
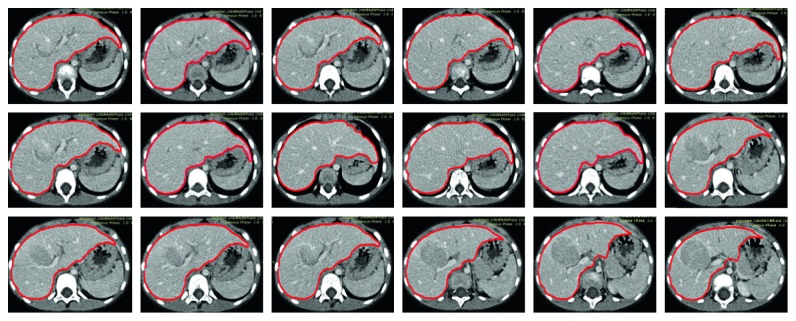
Segmentation results of some typical images in the first case, the proposed method.

**Figure 5:**
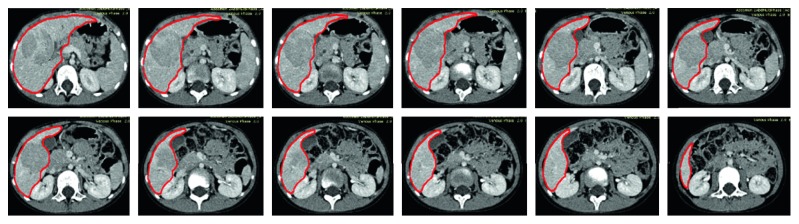
Segmentation results of some typical images in the second case, the proposed method.

**Figure 6:**
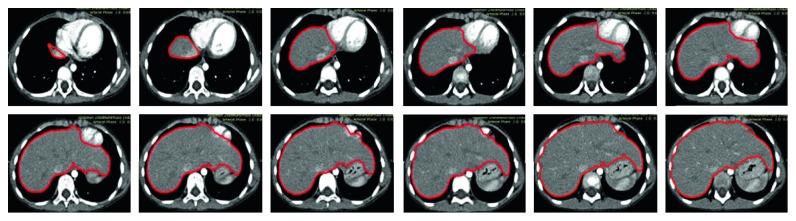
Segmentation results of some typical images in the third case, the proposed method.

**Figure 7:**
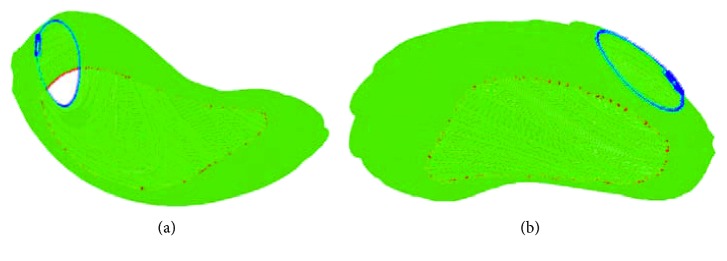
The reconstructed three-dimensional liver: (a) view 1; (b) view 2.

Since the shapes of the liver and the surrounding organs also vary significantly in different slices, the number of the pixel classes in different slices varies accordingly. The organ that is adjacent to the liver is not fixed, and it might be the stomach, the kidney, or the heart. We thus define the liver segmentation into three cases depending on its adjacent organ. In the first case, there is only stomach adjacent to the liver. In the second case, there is kidney adjacent to the liver. In the third case, there is heart adjacent to the liver. In different cases, the number of the pixel classes is adjusted automatically based on the detected thresholds by the slope difference distribution. Then, the five core image processing algorithms are applied one by one. The optimal values of the input parameters are determined for different cases, respectively. After the optimal parameters are calibrated by trial and analysis, they will be used for segmenting the livers in similar CT datasets. For the CT datasets with significant differences, the input parameters should be recalibrated again. As a result, it might require significant manual intervention to determine the optimal parameters for a specific-type CT dataset before the proposed method is run fully automatically for the whole CT dataset. The average time to process one CT image is 2.39 seconds in MATLAB with the i7-6700 CPU. Some typical liver segmentation results for the first case are shown in Figure 4. Some typical liver segmentation results for the second case are shown in Figure 5. Some typical liver segmentation results for the third case are shown in Figure 6. As can be seen, all the segmentation results are acceptable for clinical usage. With all the segmented two-dimensional liver from different slices, the whole three-dimensional liver is reconstructed by the method described in the above section.  Figure 7 shows the reconstructed three-dimensional liver.

**Figure 8:**
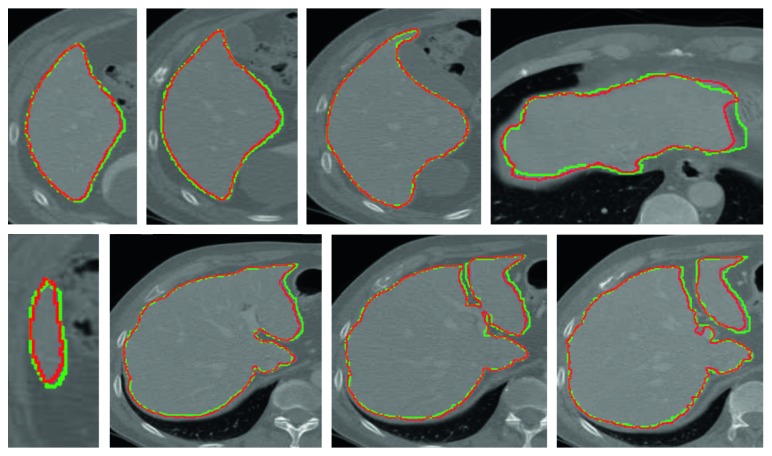
Segmentation results of some typical images from the first public dataset (the red contour denotes the automatically identified boundary, and the green contour denotes the manually identified boundary).

We use two public datasets to evaluate the proposed method further. The first public dataset (https://eee.deu.edu.tr/moodle/mod/page/view.php?id=7872) is liver transplantation donor database that provided by Emre Kavur from Dokuz Eylul University with the permission of Dokuz Eylul University Hospital. In this set, there are 20 upper abdominal CT image series that belongs to different patients and six sets are training sets. Only ground truths of the training sets are provided. Since the proposed method does not require training, we use the provided training sets to evaluate our method. The computed mean ratios of the volumetric overlap error (VOE), relative volume difference (RVD), average symmetric surface distance (ASD), root mean square symmetric surface distance (RMSD), and maximum symmetric surface distance (MSSD) were 9.6 ± 2.2%, 4.2 ± 2.5%, 1.7 ± 0.9 mm, 2.4 ± 1.1 mm, and 9.2 ± 3.1 mm, respectively. We also show some qualitative results in Figure 8. As can be seen, the automatic identified boundary of the liver matches the manually identified boundary well.

The second public dataset, 3D-IRCADb01 (https://www.ircad.fr/research/3d-ircadb-01/), consists of 20 CT scans with corresponding ground truth provided by IRCAD, the French Research Institute against Digestive Cancer. We show the quantitative comparisons with state-of-the-art methods [22–25] in Table 1. As can be seen, the comparisons are favorable. The proposed method does not require any work for training as state-of-the-art methods [22–25] do while the achieved accuracy is similar or better.

The accuracy of the proposed method differs significantly across different CT liver datasets because the proposed method is heavily relying on the accuracy of threshold selection. Yet, the optimal accuracy of slope difference-based threshold selection could not be guaranteed currently during unsupervised segmentation. In the near future, we will research on algorithms to make the slope difference-based threshold selection method be capable of always selecting optimal thresholds without supervision.

## 4. Conclusions

In this paper, a heuristic approach is proposed to segment the liver in CT images fully automatically. It calculates multiple thresholds simultaneously based on the slope difference distribution and then segment the CT image into different meaningful regions with these automatically calculated thresholds. The liver is segmented robustly with the constraint of the surrounding organs or tissues segmented beforehand. To reduce the noise, Gibbs distribution is utilized to minimize the global energy. A morphological filter is proposed to remove the small interference blobs. A morphological merging method is also proposed to unite the divided parts. Experimental results verified all the proposed image processing algorithms and the proposed approach. Since the proposed approach is very efficient and does not require any training datasets, it is thus very promising for clinical usage.

## Figures and Tables

**Table 1:** Comparison of the proposed method with state-of-the-art methods on the 3Dircadb1 dataset.

Methods	VOE (%)	RVD (%)	ASD (mm)	RMSD (mm)	MSSD (mm)
[22]	12.99 ± 5.04%	5.66 ± 5.59%	2.24 ± 1.08	NA	25.74 ± 8.85
[23]	NA	3.62 ± 5.50%	1.94 ± 1.10	4.47 ± 3.30	34.60 ± 17.70
[24]	8.74 ± 2.37%	2.41 ± 1.71%	1.45 ± 0.37	2.55 ± 0.59	26.91 ± 7.72
[25]	15.6 ± 4.3%	5.8 ± 3.5%	2.0 ± 0.9	2.9 ± 1.5	7.1 ± 6.2
Proposed	10.25 ± 4.21%	4.7 ± 3.37%	1.84 ± 0.93	2.67 ± 1.1	15.23 ± 9.67

## Data Availability

The data used to support the findings of this study are available from the corresponding author upon request.
